# Supplementary material for: Context-dependent serotonin signaling links dietary quality to foraging decisions
Source: Nat Commun. 2025 Nov 25;16:10479. doi: 10.1038/s41467-025-65491-8 (PMC12647731; doi:10.1038/s41467-025-65491-8)
Supplement: Supplementary file 1 — Supplementary Information [file 41467_2025_65491_MOESM1_ESM.pdf]

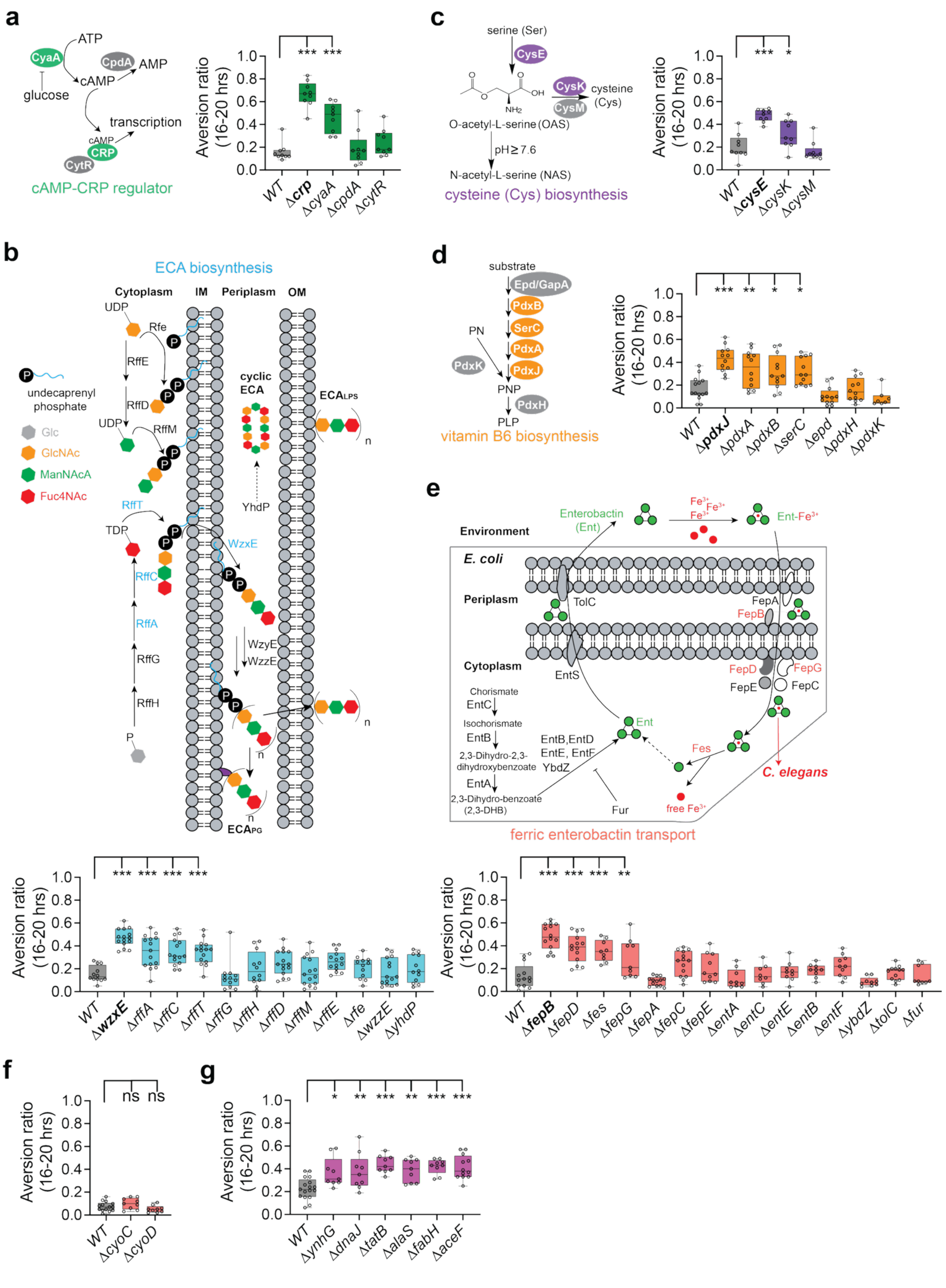

a
c
b
d
e
f
g
cAMP-CRP regulator
cysteine (Cys) biosynthesis
ECA biosynthesis
vitamin B6 biosynthesis
ferric enterobactin transport
Aversion ratio (16-20 hrs)

Supplementary Fig. 1: *C. elegans* behavioral responses to *E. coli* mutants identified from the genome-wide screen as well as other genes from the same pathways. Related to Fig. 1 and Table 1.

(**a-e**) Complete metabolic pathways and behavioral responses to additional *E. coli* mutants in each pathway, expanded from Fig. 1d (n=9-15 assays). (**f**) Behavioral responses to additional ROS-producing *E. coli* mutants (n=9-14 assays). (**g**) Behavioral responses to additional *E. coli* mutants from Table 1. Each data point indicates individual assay (n=9-17). Results are shown with median ± quartiles in boxes and Min to Max whiskers. ns, not significant, *P<0.05, **P<0.01, ***P<0.001 by one-way ANOVA corrected by Dunnett's multiple comparisons. Statistical analysis with significance is annotated; unannotated entries are not significantly different from WT. All sample sizes, statistical test used, and exact P values are provided in Supplementary Data 4. Source data are provided as a Source Data file.

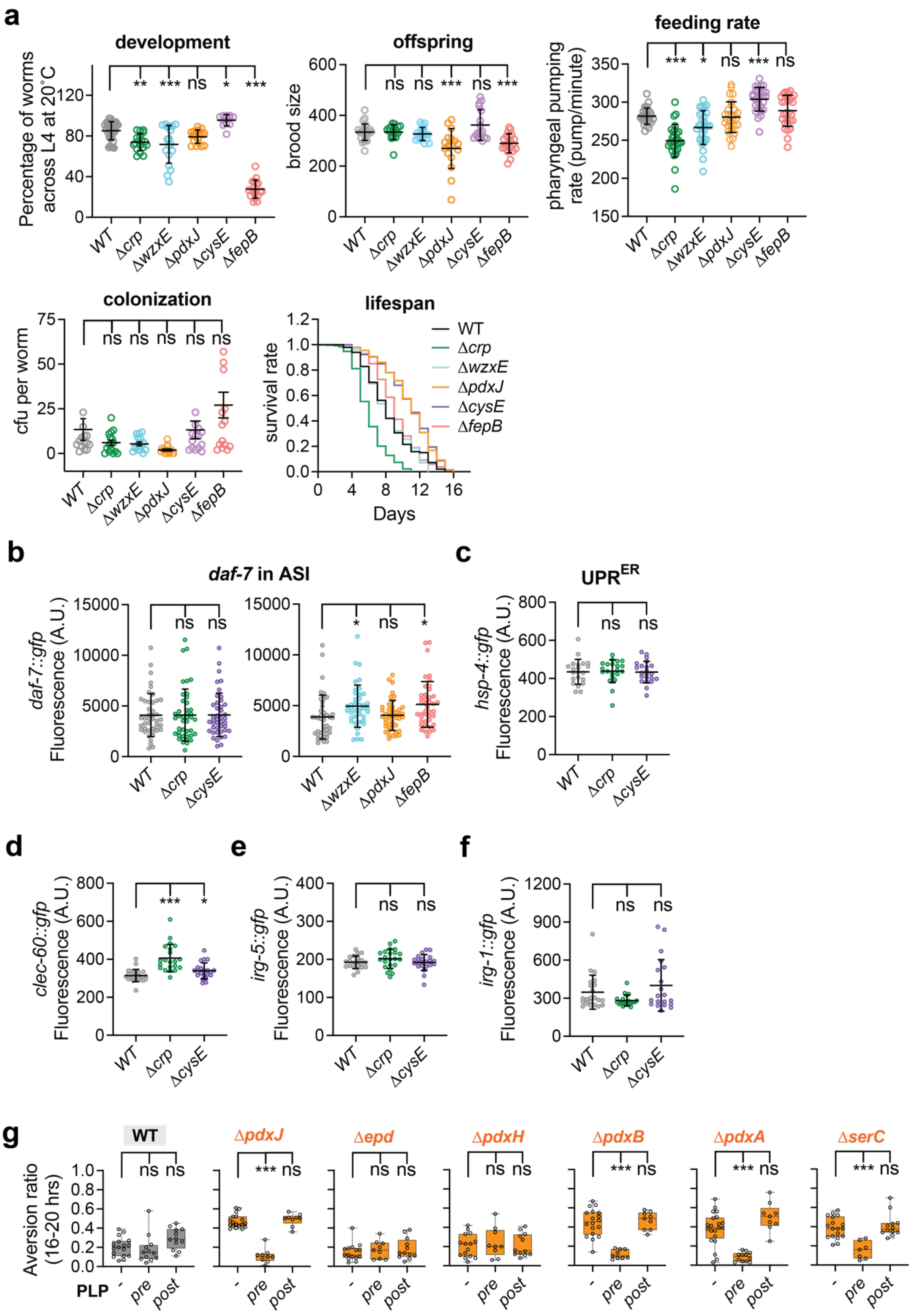
a
development
Percentage of worms across L4 at 20°C
** *** ns * ***
WT Δcrp ΔwzxE ΔpdxJ ΔcysE ΔfepB
offspring
brood size
ns ns *** ns ***
feeding rate
pharyngeal pumping rate (pump/minute)
*** * ns *** ns
colonization
cfu per worm
ns ns ns ns ns
lifespan
survival rate
Days
WT
Δcrp
ΔwzxE
ΔpdxJ
ΔcysE
ΔfepB
b
daf-7 in ASI
daf-7::gfp Fluorescence (A.U.)
ns ns
* ns *
c
UPR^ER
hsp-4::gfp Fluorescence (A.U.)
ns ns
d
clec-60::gfp Fluorescence (A.U.)
*** *
e
irg-5::gfp Fluorescence (A.U.)
ns ns
f
irg-1::gfp Fluorescence (A.U.)
ns ns
g
WT
ΔpdxJ
Δepd
ΔpdxH
ΔpdxB
ΔpdxA
ΔserC
Aversion ratio (16-20 hrs)
PLP
- pre post

Supplementary Fig. 2: Effects of mediocre diets on animal physiology. Related to Fig. 2.
(**a**) Effects of bacterial diets on *C. elegans* development, brood size, feeding rate, colonization and lifespan. For brood size (offspring), each data point represents the number of progeny from an individual animal (n=17-26). For colonization, each data point represents the number of bacteria isolated from an individual animal (n=15-16). ns, not significant, ***P<0.0001 by one-way ANOVA corrected by Dunnett's multiple comparisons. For feeding rate and development, three biological assays were conducted per condition, including at least five replicates per assay. Each data point indicates individual replicate (n=12-34). Error bars indicate mean ± SD. ns, not significant, *P<0.05, **P<0.01, ***P<0.001 by one-way ANOVA corrected by Dunnett's multiple comparisons. Lifespan assay was performed at 25 ˚C (n=6-12). (**b-f**) Effects of bacterial diets on *C. elegans* stress reporters, including *daf-7::GFP* expression in ASI (**b**, n=41-45), UPR^{ER} (*hsp-4::gfp*) (**c**, n=21-22), and the immune-response genes *clec-60::gfp* (**d**, n=21-24), *irg-5::gfp* (**e,** , n=21-22), and *irg-1::gfp* (**f**, n=20-22). GFP fluorescence intensities were quantified using Arbitrary Units (A.U.). Each data point indicates reporter expression in one animal. Error bars indicate mean ± SD. ns, not significant, *P<0.05, ***P<0.001 by One-Way ANOVA corrected by Dunnett's multiple comparisons. (**g**) Chemical supplementation of *E. coli* mutants defective in vitamin B6 synthesis with pyridoxal 5' phosphate (PLP) rescued aversion behavior when PLP was provided during bacterial growth (pre) but not when PLP was provided during the behavioral assay (post). 40 µM PLP was used for all experiments. Each data point indicates individual assay (n=7-21). Results are shown with median ± quartiles in boxes and Min to Max whiskers. ns, not significant, ***P<0.001 by One-Way ANOVA, corrected by Dunnett's multiple comparisons. All sample sizes, statistical test used, and exact P values are provided in Supplementary Data 4.
Source data are provided as a Source Data file.

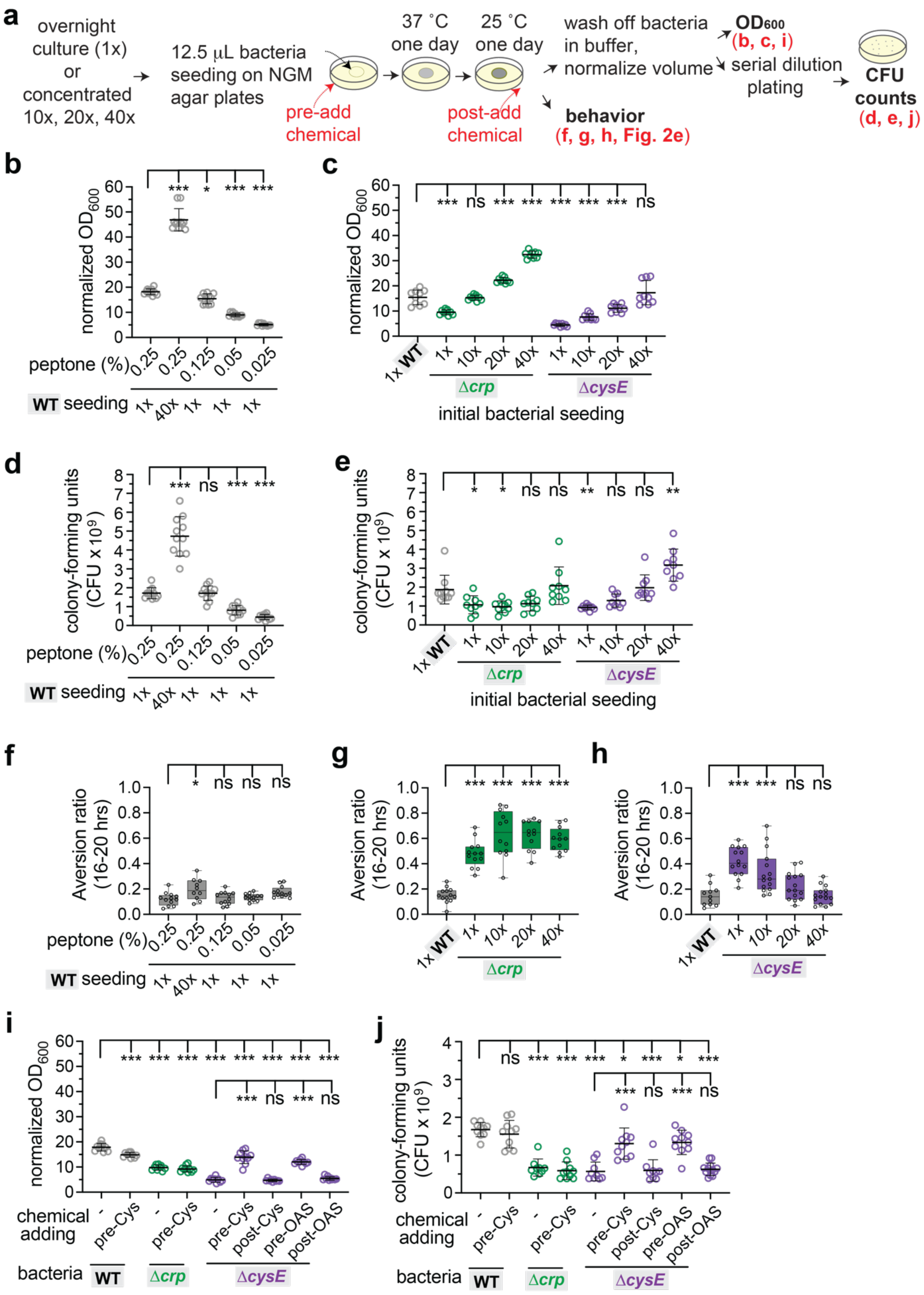
a
overnight culture (1x) or concentrated 10x, 20x, 40x
12.5 μL bacteria seeding on NGM agar plates
pre-add chemical
37 °C one day
25 °C one day
post-add chemical
wash off bacteria in buffer, normalize volume
OD600 (b, c, i)
serial dilution plating
CFU counts (d, e, j)
behavior (f, g, h, Fig. 2e)
b
normalized OD600
peptone (%) 0.25 0.25 0.125 0.05 0.025
WT seeding 1x 40x 1x 1x 1x
*** * *** ***
c
normalized OD600
*** ns *** *** *** *** *** ns
1x WT 1x 10x 20x 40x 1x 10x 20x 40x
Δcrp
ΔcysE
initial bacterial seeding
d
colony-forming units (CFU x 10^9)
*** ns *** ***
peptone (%) 0.25 0.25 0.125 0.05 0.025
WT seeding 1x 40x 1x 1x 1x
e
colony-forming units (CFU x 10^9)
* * ns ns ** ns ns **
1x WT 1x 10x 20x 40x 1x 10x 20x 40x
Δcrp
ΔcysE
initial bacterial seeding
f
Aversion ratio (16-20 hrs)
* ns ns ns
peptone (%) 0.25 0.25 0.125 0.05 0.025
WT seeding 1x 40x 1x 1x 1x
g
Aversion ratio (16-20 hrs)
*** *** *** ***
1x WT 1x 10x 20x 40x
Δcrp
h
Aversion ratio (16-20 hrs)
*** *** ns ns
1x WT 1x 10x 20x 40x
ΔcysE
i
normalized OD600
*** *** *** *** *** *** *** ***
*** ns *** ns
chemical adding - pre-Cys - pre-Cys - pre-Cys post-Cys pre-OAS post-OAS
bacteria WT Δcrp ΔcysE
j
colony-forming units (CFU x 10^9)
ns *** *** *** * *** * ***
*** ns *** ns
chemical adding - pre-Cys - pre-Cys - pre-Cys post-Cys pre-OAS post-OAS
bacteria WT Δcrp ΔcysE

Supplementary Fig. 3: Effects of bacterial density on aversion behavior. Related to Fig. 2. (**a**) Workflow to determine bacterial OD_{600}, colony-forming units (CFU) and behavioral responses (n=9-12 assays). A subset of bacterial counts in (**b-e**) and matching behavioral responses (**f-h**) are summarized in **Fig. 2e**; additional bacterial counts in (**i, j**) correspond to behavioral responses after cysteine supplementation in **Fig. 2e**. All assay plates in panels (**c, e**, **i, j**) contained 0.25% peptone. For bacterial counts, results are shown with mean ± SEM. ns, not significant, *P<0.05, **P<0.01, ***P<0.001 by one-way ANOVA corrected by Dunnett's multiple comparisons. Note that the large *Δcrp* bacteria have a higher OD_{600} relative to cell number.

For behavioral assays (**f, g, h**), each data point indicates individual assay (n=10-15). Results are shown with median ± quartiles in boxes and Min to Max whiskers. ns, not significant, ***P<0.001 by one-way ANOVA corrected by Dunnett's multiple comparisons. All sample sizes, statistical test used, and exact P values are provided in Supplementary Data 4. Source data are provided as a Source Data file.

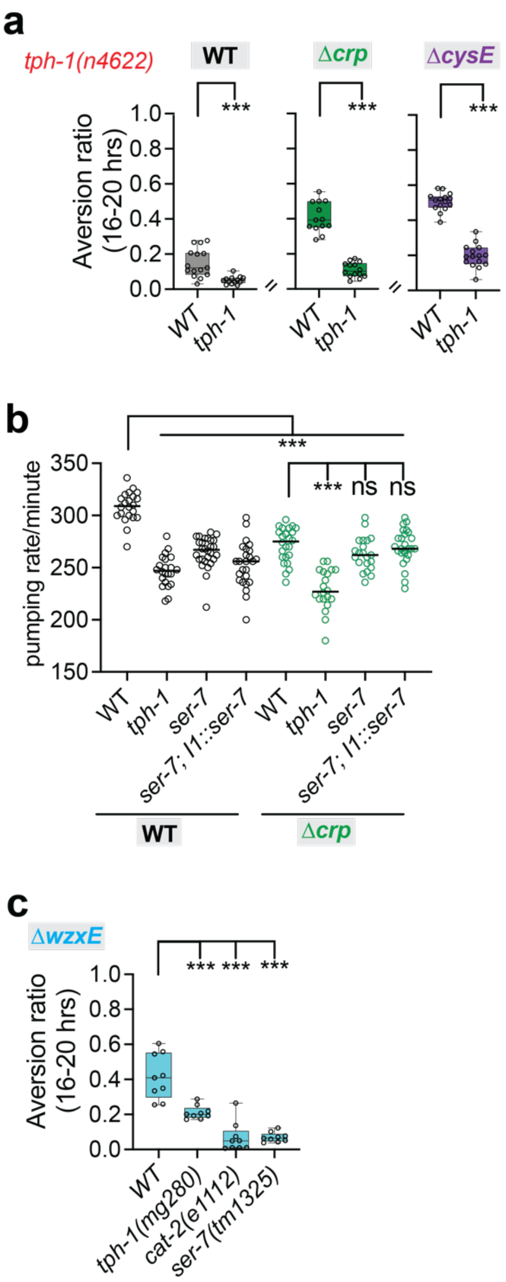

Supplementary Fig. 4: Additional analysis of serotonin and dopamine mutants on mediocre diets. Related to Figs. 3, 5, and 6.

(**a**) Aversion ratios of WT and *tph-1*(*n4622*) animals on three diets, related to **Fig. 3a** (n=13-15 assays); all other experiments used *tph-1(mg280)*. (**b**) Pharyngeal pumping (feeding) rates on wild-type and Δ*crp E. coli* of wild-type, *tph-1*(*mg280*), *ser-7(tm1325)*, and *ser-7(tm1325)* with transgenic rescue in the I1 neuron, related to **Fig. 5**. Each data point indicates feeding rate from an individual animal (n=20-28). ns, not significant, ***P<0.001 by one-way ANOVA corrected by Dunnett's multiple comparisons. (**c**) Aversion ratios of WT, *tph-1*(*mg280*), *cat-2(e1112),* and

ser-7(tm1325) animals on the mediocre Δ*wzxE* diet, related to **Fig. 6**. Each data point indicates individual assay (n=9). Results are shown with median ± quartiles in boxes and Min to Max whiskers. ***P<0.001 by One-Way ANOVA corrected by Dunnett's multiple comparisons. Full genotypes of all *C. elegans* strains are provided in Supplementary Data 3. All sample sizes, statistical test used, and exact P values are provided in Supplementary Data 4.

Source data are provided as a Source Data file.

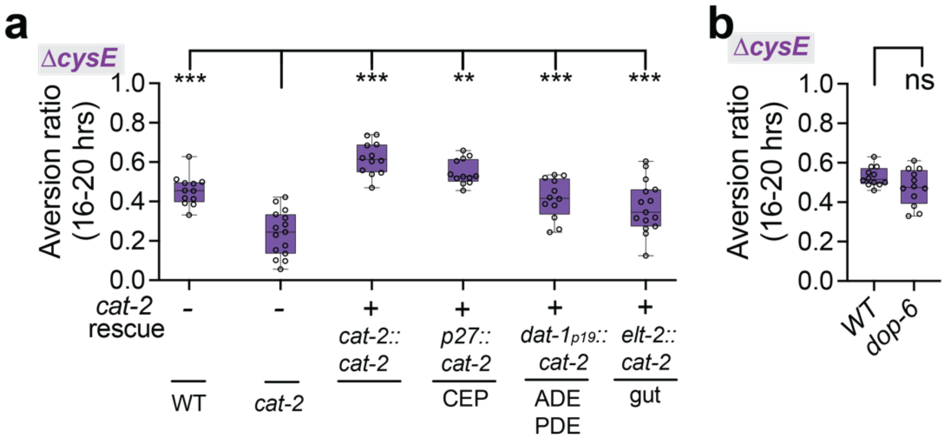

Supplementary Fig. 5: Dopamine promotes aversion to the mediocre Δ*cysE* diet. Related to Fig. 7.

Aversion ratios of *cat-2* mutants and rescue lines (**a**) or *dop-6*(*ok2090*) mutants (**b**) on the Δ*cysE* mediocre diet. Each data point indicates individual assay (n=12-15). Results are shown with median ± quartiles in boxes and Min to Max whiskers. ns, not significant, $^{**}P<0.01$, $^{***}P<0.001$ by one-way ANOVA corrected by Dunnett's multiple comparisons (panel **a**) or two-tailed, unpaired t test (panel **b**, *dop-6*). Full genotypes of all *C. elegans* strains are provided in Supplementary Data 3. All sample sizes, statistical test used, and exact P values are provided in Supplementary Data 4.

Source data are provided as a Source Data file.
