# Supplementary material for: Context-dependent serotonin signaling links dietary quality to foraging decisions
Source: Nat Commun. 2025 Nov 25;16:10479. doi: 10.1038/s41467-025-65491-8 (PMC12647731; doi:10.1038/s41467-025-65491-8)
Supplement: Supplementary file 2 — Description of Additional Supplementary Files [file 41467_2025_65491_MOESM2_ESM.pdf]

Description of Additional Supplementary Files

File Name: Supplementary Data 1
Description: Effects of chemicals on bacterial growth rates

File Name: Supplementary Data 2
Description: A full list of Reagents and Resources used in this study

File Name: Supplementary Data 3
Description: A list of full genotypes of all strains used in each figure

File Name: Supplementary Data 4
Description: Statistical table
